# Solid-State Fermentation of *Trichoderma* spp.: A New Way to Valorize the Agricultural Digestate and Produce Value-Added Bioproducts

**DOI:** 10.1021/acs.jafc.2c07388

**Published:** 2023-02-03

**Authors:** Daniela Bulgari, Carlotta Alias, Gregorio Peron, Giovanni Ribaudo, Alessandra Gianoncelli, Salvatore Savino, Houda Boureghda, Zouaoui Bouznad, Eugenio Monti, Emanuela Gobbi

**Affiliations:** † Agri-Food and Environmental Microbiology Platform, Department of Molecular and Translational Medicine, University of Brescia, Viale Europa, 11, 25123Brescia, Italy; ‡ B+LabNet-Environmental Sustainability Lab, University of Brescia, Via Branze 45, 25123Brescia, Italy; § Proteomics Platform, AgroFood Lab, Department of Molecular and Translational Medicine, University of Brescia, Viale Europa, 11, 25123Brescia, Italy; ∥ Unit of Biotechnology, Department of Molecular and Translational Medicine, University of Brescia, Viale Europa 11, 25123Brescia, Italy; ⊥ Department of Botany, Laboratory of Phytopathology and Molecular Biology, Ecole Nationale Supérieure Agronomique (ENSA), El Harrach, Algiers16200, Algeria

**Keywords:** fungal biomass, cellulase, esterase, biorefinery, citric acid, malic acid, secondary metabolites, oxylipin, gibberellin, biostimulants

## Abstract

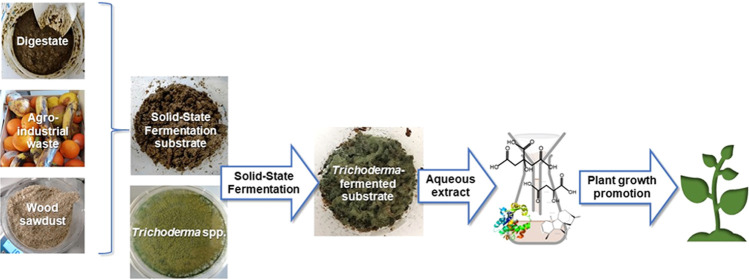

In this study, the agricultural digestate from anaerobic biogas production mixed with food wastes was used as a substrate to grow *Trichoderma reesei* RUT-C30 and *Trichoderma atroviride* Ta13 in solid-state fermentation (SSF) and produce high-value bioproducts, such as bioactive molecules to be used as ingredients for biostimulants. The *Trichoderma* spp. reached their maximum growth after 6 and 3 SSF days, respectively. Both *Trichoderma* species were able to produce cellulase, esterase, and citric and malic acids, while *T. atroviride* also produced gibberellins and oxylipins as shown by ultraperformance liquid chromatography with quadrupole time-of-flight mass spectrometry (UPLC-QTOF-MS) profiling. Experimental evaluation of germination parameters highlighted a significant promotion of tomato seed germination and root elongation induced by *T. atroviride* crude extracts from SSF. This study suggests an innovative sustainable use of the whole digestate mixed with agro-food waste as a valuable substrate in fungal biorefineries. Here, it has been applied to produce plant growth-promoting fungi and bioactive molecules for sustainable agriculture.

## Introduction

The European Union (EU) Bioeconomy Strategy^1^ highlighted the need for the sustainable conversion of both primary and waste organic resources into food, feed, and bioenergy as well as bio-based products. Anaerobic digestion (AD) is an emerging technology combining a sustainable management of organic wastes for the reduction of the greenhouse gas emission with biofuel production. The biogas production through AD generates one hundred and eighty million tonnes of residue digestate every year in 28 countries of the EU. The process is steadily increasing, boosting the number of European biomethane plants from 187 in 2011 to 725 in 2019.^2^ This massive production of digestate (the byproduct of AD) is an issue that requires the development of an environmentally sustainable and economically feasible bioprocess to valorize bioenergy wastes in the near future as also required by the Bioeconomy Strategy. Yet, the valorization and recycling of the digestate are challenges for the circular bioenergy production.

Digestate has a wide range of potential applications depending on the feedstocks of AD, the processing technology, the digestate characteristics, and the operating parameters of the process.^3^ The application as a biofertilizer or soil conditioner of the digestate derived from AD of agricultural wastes (animal manure, energy crop, straw, etc.) offers the simplest and inexpensive method but poses a threat to the quality of the receiving environment; thus, advanced applications of digestate are warranted for sustainable digestate exploitations as renewable resources.^4,5^

Filamentous fungi naturally produce and secrete a wide range of metabolites and enzymes, so they can be employed as a biocatalyst in fungal biorefineries.^6,7^ The production of hydrolytic enzymes (e.g., xylanase, chitinase, cellulase) is an example of biorefinery based on agro-industrial wastes as raw substrates. Fungi produce also a variety of secondary metabolites that are involved in their development, defense, and host-interaction.^6^ These metabolites have been an historical treasure for drug discovery; only recently, microbial value-added products have found broad applications in food (organic acids as acidulants)^8^ and agrochemical (biopesticide and biostimulant production) industries.^9,10^

Plant biostimulants are currently defined by the European Parliament as “a product stimulating plant nutrition processes independently of the product’s nutrient content with the sole aim of improving one or more of the following characteristics of the plant and its rhizosphere: nutrient use efficiency, tolerance to abiotic stress, quality traits, and availability of confined nutrients in soil or rhizosphere”.^11^ They mainly include protein hydrolysates and other N-containing compounds, seaweed extracts, chitosan, humic and fulvic acids, plant growth-promoting bacteria, and fungi.^12−14^

Only a couple of methods for the valorization of digestate as a substrate for the solid-state fermentation (SSF) have been reported up to now: as a biopesticide, with the inoculum of *Bacillus thuringiensis* (Bt)^15^ or for *Trichoderma* spp. biomass production.^16^ In SSF, microorganisms grow on a solid substrate in the absence or near absence of free water and convert the substrate into a value-added product. In the recent years, SSF has gained new attention from scientists and industries for its potential in circular bioeconomy. Microbial cultivation on waste-based substrates, to accomplish the dual benefits of complete utilization of wastes and production of value-added products such as enzymes or secondary metabolites, is one of the principles of biorefinery.^9^ The most widely used substrates are agro-industrial wastes such as wheat bran, spent coffee ground, rice husk, and bagasse.^8,10^ These substrates are easily available with low or no economical cost, and they are able to support microbial growth.

*Trichoderma* is a fungal genus including around 375 species colonizing various ecological niches ranging to soil, root, or leaf.^17^*Trichoderma* species are well known for the production of commercial enzymes, for plant growth abilities and biocontrol of plant disease, making them promising for identification and production of bioactive molecules.^7^ Many *Trichoderma* species, such as *Trichoderma harzianum*, *Trichoderma asperellum*, *Trichoderma atroviride*, *Trichoderma koningii*, *Trichoderma virens*, *Trichoderma viride*, and *Trichoderma reesei*, are commercially used as potent biocontrol agents^18^ and for cellulase production^19^ worldwide. Little is known about the possibility to produce bioactive molecules or enzymes developing a digestate-based biorefinery.

This study exploits the results of our previous study on *Trichoderma* spp. cultivation on digestate^16^ by evaluating the contextual production of value-added bioproducts that can be employed as biostimulants, through SSF of digestate added with other organic wastes (fruits and wood discards).

## Materials and Methods

### Fungal Species and Chemicals

*T. reesei* RUT-C30 (ATCC 56765) was purchased from ATCC (Manassas, VA) and *T. atroviride* Ta13 (MUT 6701) was isolated from wheat seeds (Algeria) and deposited at the Mycotheca Universitatis Taurinensis (MUT, Turin, Italy).

2,6-Dimethoxyphenol, 3,5-dinitrosalicylic acid, 4-nitrophenyl butyrate, acetonitrile, bovine serum albumin, carboxymethyl cellulose, citrate solution, citric acid, formic acid, malic acid, methanol, Na acetate buffer, Na phosphate buffer, Na phosphate–citrate, potato dextrose agar, trichloroacetic acid, Tris-HCl buffer, and Triton X-100 were purchased from Merck (Darmstadt, Germany).

### Digestate

The digestate was collected from a full-scale biomethane plant operating in the Lombardy Region (northern Italy). The anaerobic digestion input was a mix of manure and vegetables as reported in Table S1. Samples of whole digestate (liquid and solid phases) were collected at the end of the AD and stored at −20 °C until use.

### Substrate Preparation and Solid-State Fermentation

Apple, banana, and grape fruits no longer suitable for consumption were ground in a blender (Waring commercial, McConnellsburg) to obtain particles of 1–2 mm of diameter. The whole digestate was thawed and manually mixed with shredded fruits in a ratio of 70:30 w/w. The substrate composition is reported in Table 1. Wood sawdust, a local carpentry byproduct, was added to the whole digestate to reduce the free water and adjust the moisture content to 73%. SSF was carried out in micropropagation boxes equipped with a 0.45 μm filter (Microbox, Micropoli, Italy), each containing 70 g of substrate. Filled boxes were subjected to two consecutive cycles of sterilization (121 °C for 15 min each). For the analyses, seven time points were considered: T0, T1, T2, T3, T4, T5, and T6 corresponding to 0, 3, 6, 13, 20, 27, and 34 days after inoculation. *T. reesei* RUT-C30 and *T. atroviride* Ta13 were routinely grown on slants of PDA at 26 °C. Then, 100 μL of conidia suspension (10^6^ in sterile distilled water) was inoculated in each box; four replicate boxes were produced for each time point and for each strain. In addition, four replicate boxes were not inoculated and served as a control (SSF-NI). All of the boxes were incubated at 26 °C and 60% relative humidity (RH) under illumination of 12 h light/12 h dark cycles, using daylight tubes 24 W/m^2^, in a climatic chamber (model 720, Binder) for 34 days. For each time point, and for each strain, one replicate was used for fungal biomass quantification and stored at −80 °C until use. The remaining three replicates were treated as described below to obtain the crude extract for enzymatic and metabolomic analyses.

**Table 1:** Constituents of the Culture Substrate for Solid-State Fermentation of *Trichoderma* spp.

components	% (w/w)	amount per culture (g)
whole digestate	70	49
apple - *Malus domestica*	10	7
banana - *Musa acuminata*	10	7
grape - *Vitis vinifera*	10	7

modifiers		
wood sawdust	∼20 of total weight	

### Fungal Biomass Quantification

The mycelial mass was quantified using the standard curve method as described by Alias et al.^16^ Briefly, 5 g of homogenized fermented substrate was collected at 0, 3, 6, 13, 20, 27, and 34 days after inoculation. Samples were ground to fine powder, and the genomic DNA was extracted from each sample. After spectrophotometric quantification, the DNA was submitted to real-time PCR amplification with the TCal primer pair.^20^ The experiment was performed in duplicate.

### Crude Extract Collection

The substrates of each box were submerged with 100 mL of sterile distilled water and stirred for 20 min. Suspensions were centrifuged at 4000*g* for 20 min, and the supernatants were stored at −20 °C until use. The experiment was performed in duplicate.

### Protease Assay

The proteolytic activity was measured by a modification of the method of Anson.^21^ To test the protease activity at different pH values, 1% (w/v) bovine serum albumin was diluted in Na acetate buffer (100 mM, pH 5), Na phosphate buffer (100 mM, pH 7), and Tris-HCl buffer (100 mM, pH 9). Then, 50 μL of crude extract was added to 500 μL of each substrate solution and incubated at 37 °C for 2, 4, and 24 h. The reaction was stopped by adding 500 μL of 10% (w/v) prechilled trichloroacetic acid (TCA); the mixture was cooled on ice, and the test tubes were centrifuged at 20,000*g* for 10 min at 4 °C to remove the unhydrolyzed protein. The absorbance of the clear supernatant solution was measured at 280 nm using an ultraviolet–visible (UV–vis) microplate reader (EnSight multimode reader, PerkinElmer Waltham). One unit (U) was arbitrarily defined as the amount of enzyme that produces an increase in absorbance of 0.001 per minute under the assay condition,^22,23^ measured as the quantity of TCA-soluble products.^24^

### Laccase Activity Assay

The laccase activity was determined with 2,6-dimethoxyphenol (DMP) as a substrate.^25^ To test the laccase activity at different pH, DMP was diluted in Na phosphate–citrate buffer (100 mM, pH 4, and pH 6) and Na phosphate buffer (100 mM, pH 8). The crude extract (40 μL) was added to 160 μL of DMP (2.5 mM). The oxidation of DMP was spectrophotometrically determined by continuously recording the absorbance at 468 nm at 35 °C for 120 min. One unit (U) was arbitrarily defined as the amount of enzyme that produces an increase in absorbance at 468 nm of 1.0 per min at 35 °C.^24^

### Esterase Activity Assay

The esterase activity was determined spectrophotometrically by measuring the hydrolysis of 4-nitrophenyl butyrate (pNPB) as a substrate.^26^ The pNPB dissolved in acetonitrile (50 mM) was added to 200 μL of Na phosphate buffer (100 mM, pH 7.5) with 0.5% (v/v) Triton X-100 to 0.5 mM pNPB as the final concentration; 10 μL of crude extract was used as an enzyme source. The enzymatic reaction was carried out at 25 °C for 15 min, and the release of pNP was measured at 405 nm. Enzyme activity was calculated using the extinction coefficient of pNP corresponding to 18.5 mM^–1^ cm^–1^. One Unit is defined as the amount of the enzyme that catalyzes the conversion of one micromole of substrate per minute under the specified conditions of the assay method and normalized by grams of the fermented substrate (U/g). The statistical correlation between groups was analyzed by Student’s *t*-test.

### Cellulolytic Assay

The endoglucanase (EG) activity was assessed by measuring the release of reducing sugars in a reaction mixture containing the crude extract and carboxymethyl cellulose (0.5% w/v) as a substrate in 50 mM Na acetate buffer (pH 5) at 50 °C for 60 min (*T. reesei*) or 120 min (*T. atroviride*).^27^ The reducing sugars released were determined using the 3,5-dinitrosalicylic acid (DNS) method. One unit (U) of endoglucanase activity was defined as the amount of enzymes that released 1 μmol of glucose equimolar per minute under the assay conditions^27^ and normalized by grams of the fermented substrate (U/g).

### Citric Acid Determination and Organic Acid Quantification

The detection of citric and malic acids in the extracts was first performed by electrospray ionization mass spectrometry (ESI-MS), as described elsewhere.^28^ Briefly, mass spectra were recorded by direct infusion ESI on an LCQ Fleet ion trap spectrometer (Thermo Fisher Scientific). The instrument was set in negative ionization mode with 5.0 kV spray voltage, −1.0 V capillary voltage, and −40 V tube lens values. The ESI temperature was maintained at 225 °C. Gas flow rates (arb): sheat, 10; aux, 0; and sweep, 0. The infusion flow rate was set to 5 μL/min. Samples were diluted in methanol (1:1000) before acquisition. For data processing, Qual Browser Thermo Xcalibur 4.0.27.13 software (Thermo Fisher Scientific) was used. Quantification of organic acids (citric and malic acids) was performed by ultraperformance liquid chromatography (UPLC) coupled to a quadrupole time-of-flight (QTOF) analyzer, using the same instrumentation and method described below for primary and secondary metabolite profiling. A calibration curve of standard citrate solutions was built in the range of 0.04–80 μg/mL. The equation of the curve (*y* = 55.332*x* + 17.66; *R*^2^ = 0.999) was used to calculate the concentration of citric and malic acids (semiquantification) in the extracts from the area under the curve (AUC) values of their respective peaks.

### Profiling of Primary and Secondary Metabolites in Fermented Substrates

Chemical profiling of aqueous extracts of the fermented substrates was performed using UPLC-QTOF-MS. The instrument consisted of a Waters Acquity UPLC system coupled to a Waters Xevo G2 QTOF MS detector, operating in ESI (−) mode. For the chromatographic separation, an Agilent Eclipse plus C18 column (2.1 mm × 50 mm, 1.8 μm) was used as a stationary phase and a gradient mixture of acetonitrile (A) and 0.1% formic acid in water (B) was used as a mobile phase. The gradient was as follows: 0 min, 2% A; 1 min, 2% A; 7.5 min, 85% A; 10 min, 100% A; 12 min, 100% A; 12.5 min, 2% A; and isocratic up to 14 min. The flow rate was 0.35 mL/min. MS parameters were as follows: sampling cone voltage, 40 V; source offset, 80 V; capillary voltage, 3,500 V; nebulizer gas (N_2_) flow rate, 800 L/h; and desolvation temperature, 450 °C. The mass accuracy and reproducibility were maintained by infusing lock mass (leucine-enkephalin [M – H]^−^= 554.2620 *m*/*z*) through Lockspray at a flow rate of 20 μL/min. Centroid data were collected in the *m*/*z* range of 50–1200, and the *m*/*z* values were automatically corrected during acquisition using lock mass. MS^e^ data were also collected to obtain information about the fragmentation of detected compounds, applying a collision voltage of 30 V. For data processing, MassLynx V4.2 software (Waters) was used. Accurate *m*/*z* values of parent ions and fragments were then used to perform tentative identification of metabolites, by comparison with open source databases (HMDB: https://hmdb.ca/; Metlin: https://metlin.scripps.edu; and Fungalmet: http://www.fungalmet.org) and available literature.

### *Lepidium sativum* and *Solanum lycopersicum* Seed Germination and Root Elongation Assay

The assay was performed according to the Italian Environmental Agency guidelines.^29^ Briefly, seeds of *L. sativum* and *S. lycopersicum* not treated with fungicides were preliminary checked for vitality in distilled water in the dark at 25 ± 1 °C (germination rates >90%). Crude extracts were tested at six doses (100, 30, 15, 7.5, 3.75, and 1.8% v/v), and distilled water was used as negative control. Three replicates per treatment were arranged by wetting a Whatman no. 1 filter paper with 2 mL of each solution. Ten seeds for each replicate were distributed on the filter and incubated at 25 ± 1 °C in the dark for 72 h (*L. sativum*) or 144 h (*S. lycopersicum*). At the end of the incubation time, complete sprouts (≥1 mm) and root lengths were evaluated to calculate the germination index (GI) using the eq. , where *S*_t_ and *S*_c_ are the mean number of germinated seeds in treatments and control samples, respectively, and *L*_t_ and *L*_c_ are the mean root length of treatments and control samples, respectively. Results were expressed as GI ± standard deviation (SD). The statistical correlation between groups was analyzed by Student’s *t*-test.

## Results

### *Trichoderma* spp. Biomass Quantification

**Figure 1:**
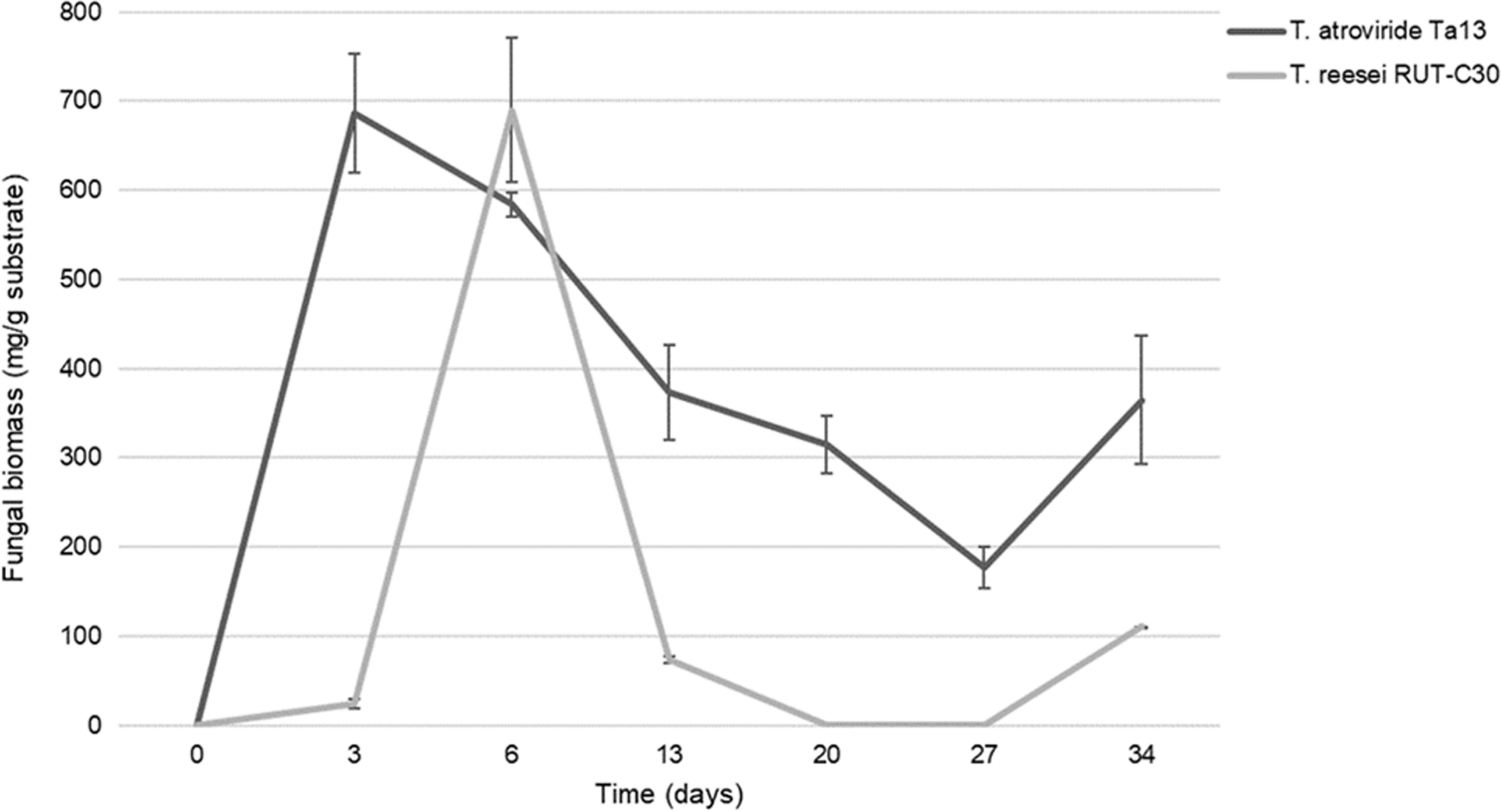
Biomass quantification of *T. reesei* and *T. atroviride* during 34 days of SSF.

The growth of *Trichoderma* spp. was monitored at different time points during 34 days of fermentation (Figure 1). The two species had different growth profiles, namely, *T. atroviride* Ta13 reached the maximum amount of mycelium rapidly, only after 3 days of culture (686.5 ± 67.1 mg/g substrate), followed by a steady decrease until the 27th day (177.3 ± 23.5 mg/g substrate). An increase of the biomass was observed at the last time point evaluated (34th day), with 364.7 ± 72.0 mg/g substrate. On the other hand, *T. reesei* RUT-C30 reached the maximum amount of mycelium later, after 6 days of culture (689.8 ± 80.5 mg/g substrate). The biomass decreased rapidly, with an undetectable amount between days 20 and 27; also this strain showed an increase of the mycelium (110.7 ± 0.5 mg/g substrate) at the end of the observational period (34th day).

### Production of Fungal Enzymes

**Figure 2:**
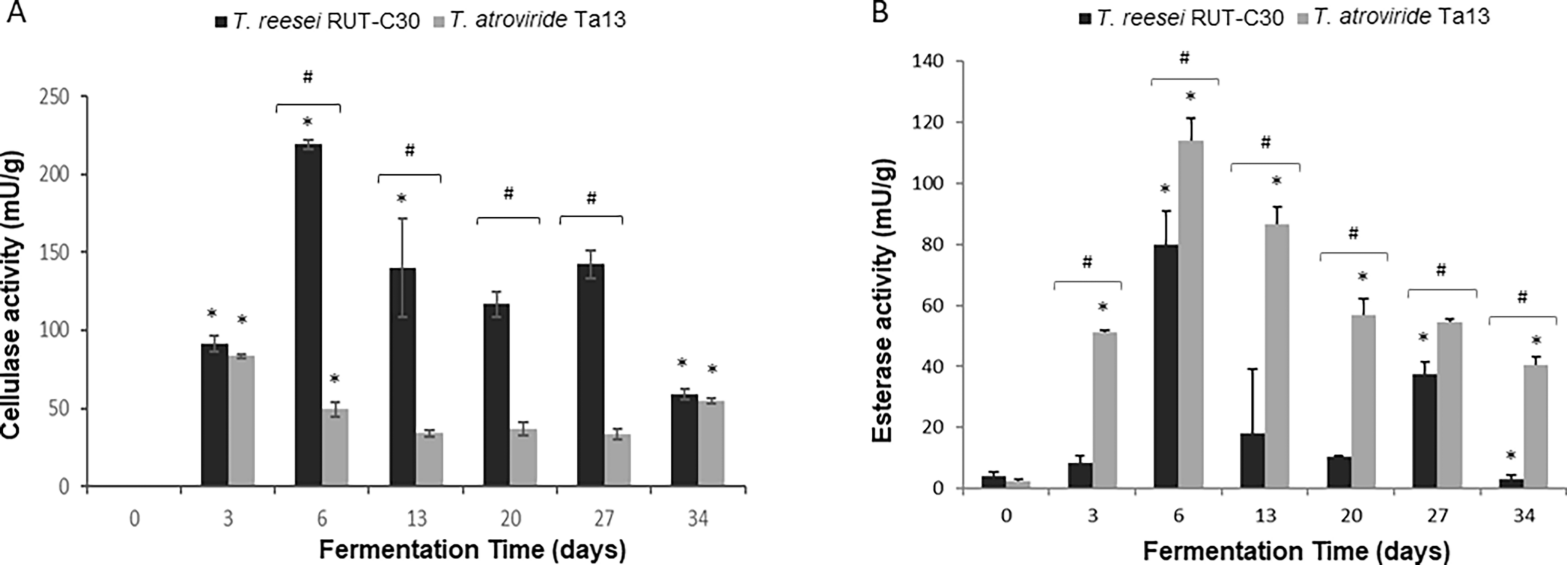
Cellulase (A) and esterase activity (B) measured in the crude extracts of *T. reesei* and *T. atroviride* at different fermentation time points. Data represent the average ± standard deviation of biological replicate cultures (*n* = 3). *p < 0.05: statistically significant in different time points according to Student’s *t*-test; #*p* < 0.05: statistically significant between *T. reesei* and *T. atroviride* at the same time point.

Protease, laccase, esterase, and cellulase activities were assessed at different time points. No protease and laccase activities were detected at any time points at any tested conditions (data not shown). Conversely, esterase and cellulase activities were measured in the crude extracts obtained from both *T. reesei* RUT-C30 and *T. atroviride* Ta13 grown on digestate-based substrates. Figure 2 shows the time course of esterase and cellulase activity in SSF. The esterase activity reached the maximum for both *T. reesei* RUT-C30 and *T. atroviride* Ta13 at 6 SSF days, and it was 113.9 ± 11.9 and 163.1 ± 7.3 mU/g, respectively. In *T. atroviride*, the activity slightly decreased, while in *T. reesei* was 4-fold reduced after 13 SSF days and successively did not follow a precise trend. The cellulase activity differed in quantity and timing among the two *Trichoderma* species. In *T. atroviride*, the highest activity was reached after 3 SSF days with the lower value of 83.3 ± 1.6 mU/g, followed by a weak decrease to 49.5 ± 4.6 mU/g, and then remained nearly constant. In *T. reesei*, the production peak was much higher (219.1 ± 3.3 mU/g) at 6 SSF days and progressively decreased to 58.9 ± 3.4 mU/g. The cellulase activity was similar at 3 SSF and 34 SSF days, while it was significantly different at the other time points.

### Organic Acid Quantification

The time courses of citric and malic acid productions in SSF are reported in Table 2. In both *T. reesei* and *T. atroviride* crude extracts, citric and malic acids were already present at 0 SSF days. The amount of citric acid in the crude extract of both fungal strains decreased at 3 SSF days and started to increase at 6 SSF days, reaching the highest value at 13 and 27 SSF days (104.8 ± 1 and 87.0 ± 0.4 μg/mL, respectively). Malic acid progressively decreased during the fermentation process, and its amounts were below the detection threshold after 20 and 6 days in *T. atroviride* and *T. reesei*, respectively.

**Table 2:** Citric and Malic Acid Production during the Solid-State Fermentation Process^a^,^b^

fermentation time		citric acid (μg/mL)		malic acid (μg/mL)	
sampling points	days	*T. reesei*	*T. atroviride*	*T. reesei*	*T. atroviride*
T0	0	91.6 ± 1.3	53.89 ± 0.5	8.73 ± 0.06	7.5 ± 0.6
T1	3	82.6 ± 0.3*	32.5 ± 0.4*	7.7 ± 0.4*	5.1 ± 0.5*
T2	6	82.7 ± 1.6*	39.2 ± 1.3*	6.9 ± 0.3*	<LOD
T3	13	104.8 ± 0.7*	61.8 ± 1.3*	6.4 ± 0.5*	<LOD
T4	20	99.8 ± 0.8*	69.5 ± 1.3*	<LOD	<LOD
T5	27	100.5 ± 1.0*	86.9 ± 0.4*	<LOD	<LOD
T6	34	<LOD	82.7 ± 0.5*	<LOD	<LOD

a Results are expressed as μg/mL of aqueous SSF extract. *p < 0.05: statistically significant compared to T0 within the species, according to Student’s *t*-test.

b LOD: limit of detection.

### Profiling of Fungal Metabolites

**Figure 3:**
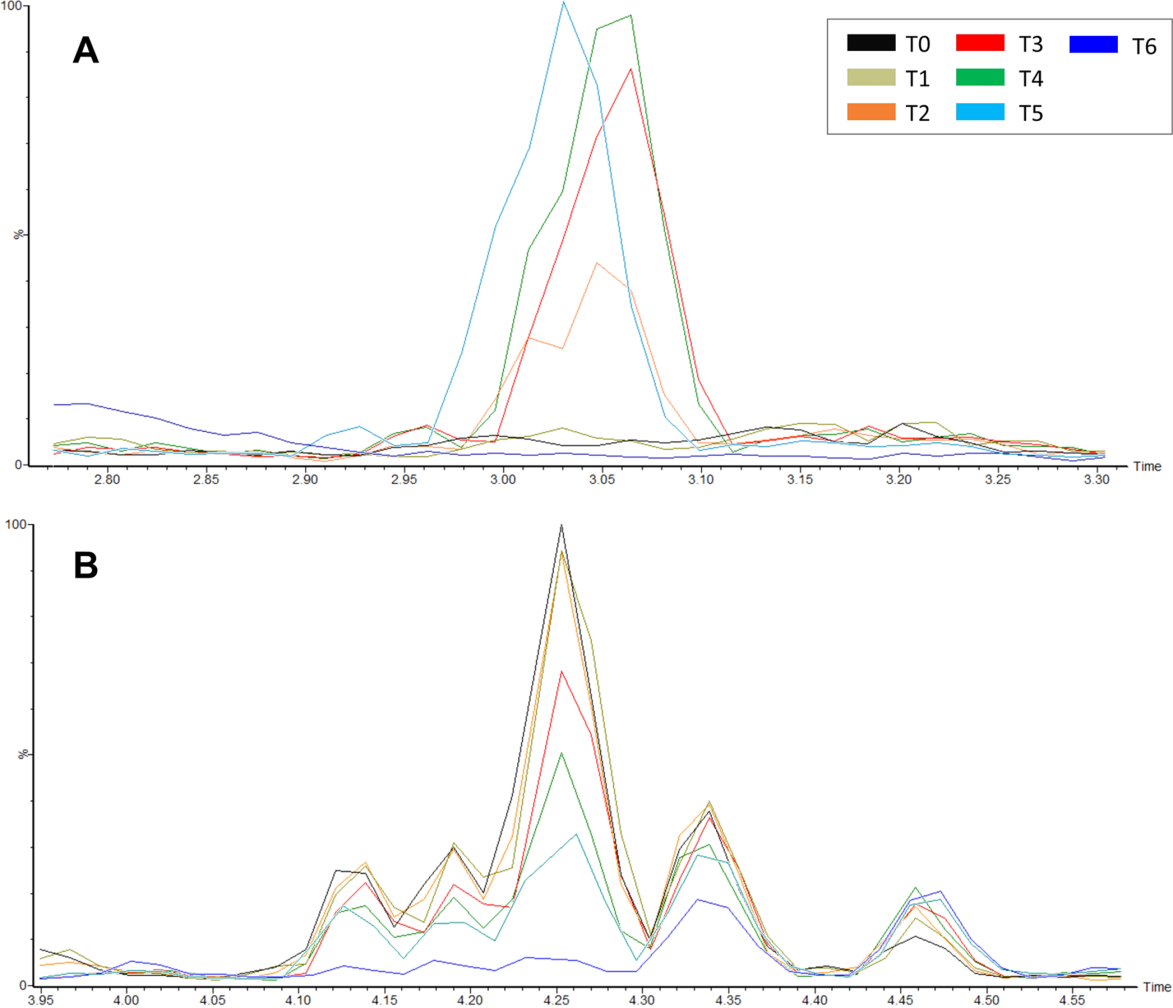
UPLC-QTOF chromatograms showing changes in the intensity of metabolites in *T. reesei* during SSF. Specifically, the peaks at 3.05 min (A) and at 4.10–4.5 min (B) are shown. For full-scale chromatograms, refer to Figure S1.

Fungal metabolites were profiled by UPLC-QTOF at six time points during the SSF process (Figures S1 and S2). The number of detected compounds as well as signal intensities changed during fermentation and between the analyzed *Trichoderma* species and the control SSF-NI (Figure S3). Specifically, in the chromatograms of *T. reesei* RUT-C30 fermented crude extract (Figures 3A and S1), a gradual increase of the AUC of the peak at 3.05 min associated to a *m*/*z* = 1415.4614 was observed. On the basis of the MS data, the metabolite could not be identified, considering also that a significant fragmentation of this molecular species was not obtained from the MS^e^ experiment. However, using the “Elemental Composition” tool of MassLynx software (Waters), a theoretical molecular formula for this metabolite was calculated (C_52_H_88_O_44_). From the evaluation of the molecular formula and retention time of the peak, we suppose that the metabolite can be a polysaccharide formed from cellulose during the SSF process. Nevertheless, further investigations are needed.

Other changes observed in the chromatograms from *T. reesei* RUT-C30 deal with a cluster of peaks between 4.1 and 4.5 min (Figure 3B and Figure S1), whose AUC gradually decreased during the SSF process. Among these, the peaks at RT = 4.19 and 4.33 min with *m*/*z* 273.0385 (theoretical molecular formula: C_14_H_10_O_6_) and 327.0852 (theoretical molecular formula: C_18_H_16_O_6_) were associated to unknown xanthones, on the basis of previous observations within the *Trichoderma* genus.^30^

**Figure 4:**
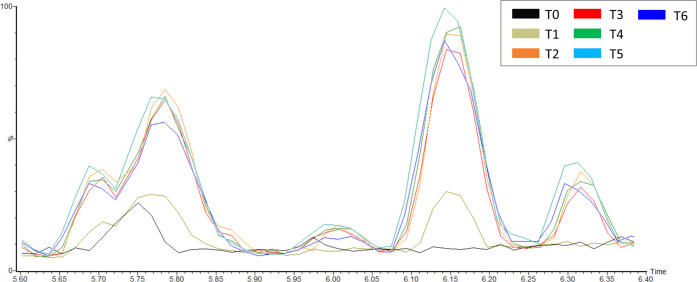
UPLC-QTOF chromatograms showing changes in the intensity of metabolites in *T. atroviride* during SSF. Specifically, peaks in the retention time range of 5.65–6.40 min are shown. For full-scale chromatograms, refer to Figure S2.

**Figure 5:**
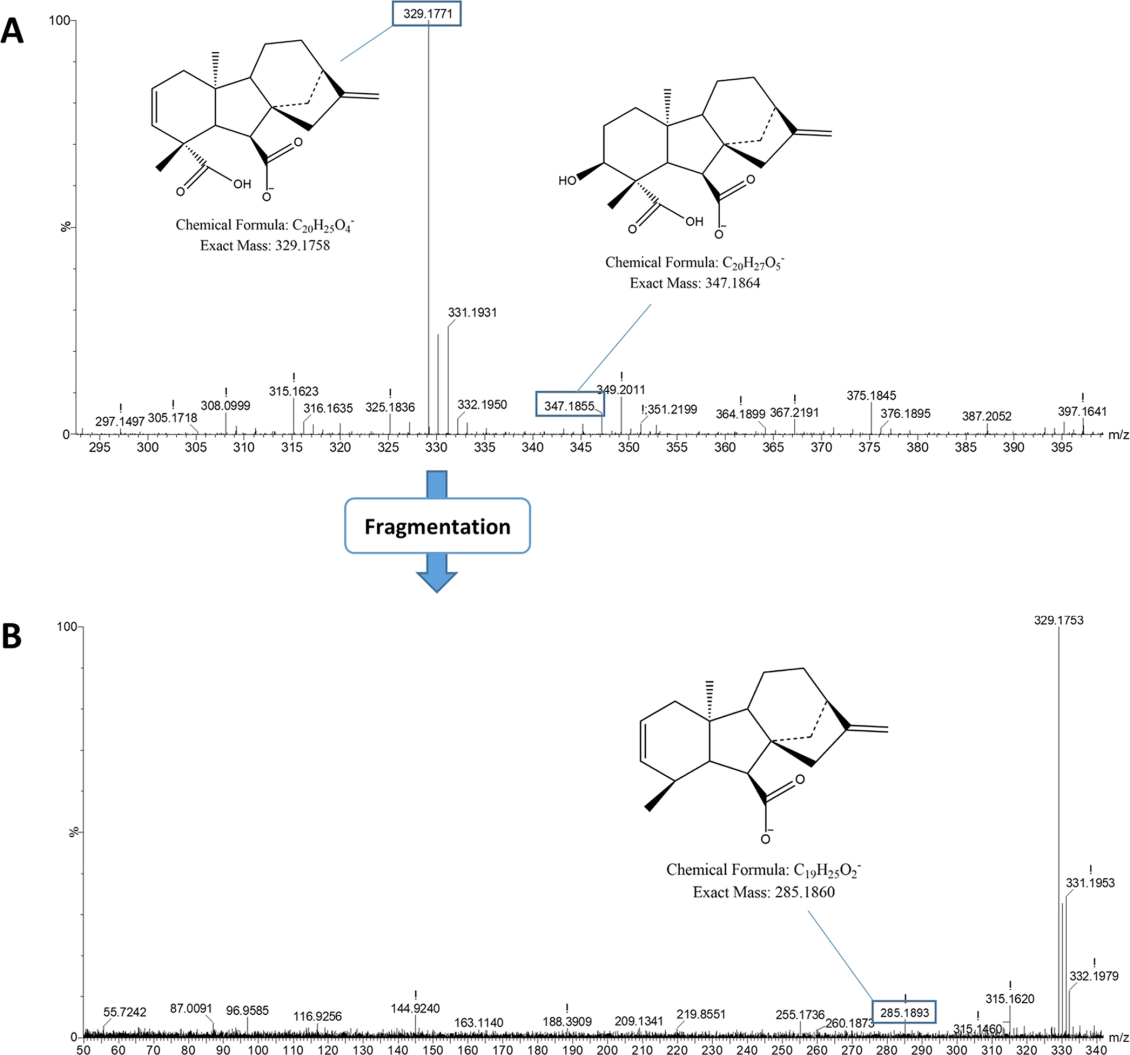
MS spectra of the molecular species eluted at 5.71 min in the UPLC-QTOF analysis of the substrate fermented with *T. atroviride*. The metabolite was tentatively identified as gibberellin A14. Panel A shows the full-scan spectrum, where the molecular ion (*m*/*z* = 347.1855) and its main fragment with *m*/*z* = 329.1771 are indicated. Panel B shows the spectrum obtained from the fragmentation (MS^e^) of the ion at *m*/*z* = 329.1771, yielding the fragment at *m*/*z* = 285.1893 after the loss of CO_2_.

**Figure 6:**
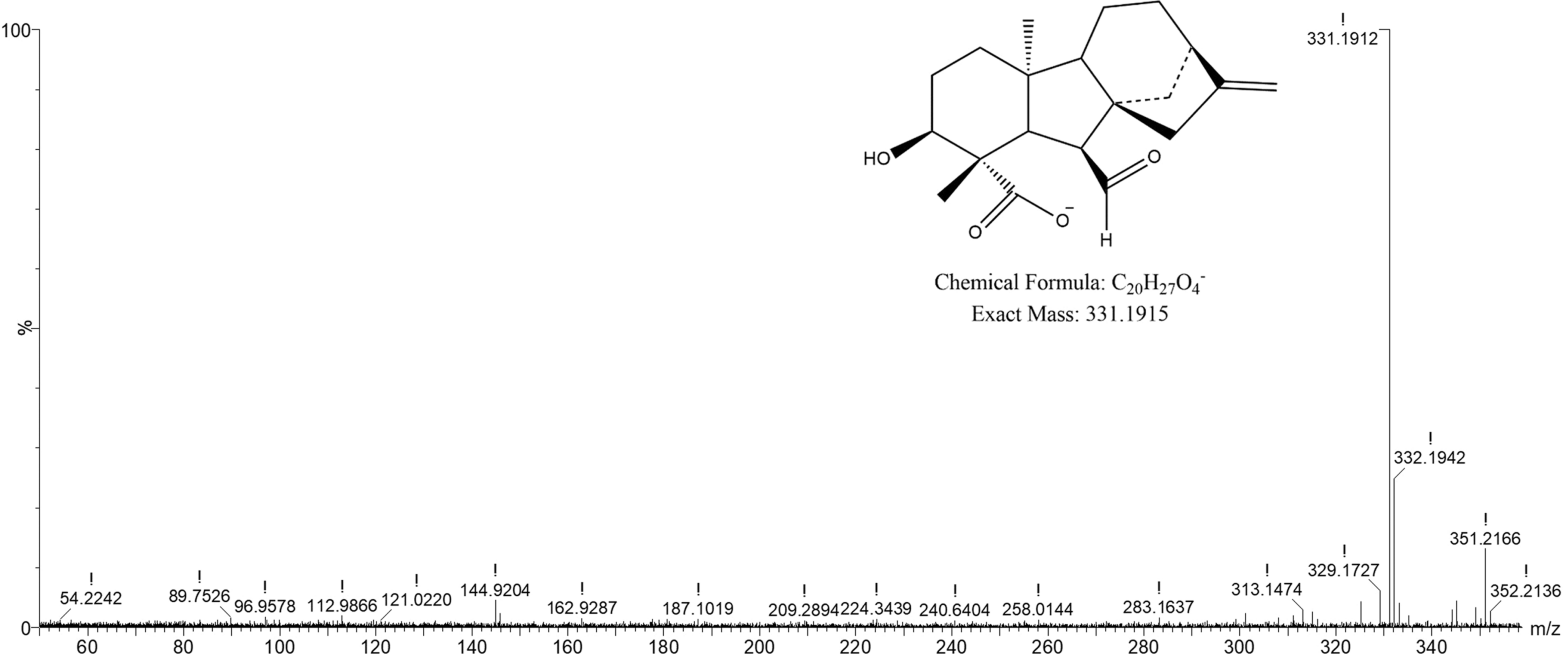
Full-scan MS spectrum of the molecular species eluted at 5.78 min in the UPLC-QTOF analysis of the substrate fermented with *T. atroviride*. The metabolite was tentatively identified as gibberellin A14 aldehyde. The molecular ion with *m*/*z* = 331.1912 is indicated.

Regarding fermentation with *T. atroviride* Ta13, chromatograms showed significant increases of the AUC of peaks between 5.71 and 6.15 min (Figures 4 and S2). Specifically, peaks at 5.71 and 5.79 min, with *m*/*z* 329.1771 (C_20_H_26_O_4_) and 331.1912 (C_20_H_28_O_4_), respectively, were tentatively identified as gibberellins. More in detail, as shown in the MS spectrum in Figure 5A, the molecular species with *m*/*z* 329.1771 was associated with the [M–H_2_O–H]^−^ fragment of the parent ion at *m*/*z* 347.1855, identified as gibberellin A14. The identification was supported by a fragment at *m*/*z* 285.1893 detected in MS2 (Figure 5B), indicating the loss of CO_2_. Regarding the species with *m*/*z* 331.1912, it was tentatively identified as gibberellin A14 aldehyde on the basis of its accurate mass (Figure 6). MS^e^ did not show any significant fragmentation that could support the identification. Another peak at 6.15 min (Figure 4), with *m*/*z* 349.2025 (C_20_H_30_O_5_) and fragments at *m*/*z* 331.1907 and 303.1974 (Figure S4, panels A and B, respectively), was associated to an oxylipin (polyhydroxylated fatty acid), on the basis of the previous literature on the presence of these compounds in the *Trichoderma* genus.^31^

### Effects of Crude Extracts on *L. sativum* and *S. lycopersicum* Germination Indexes

**Figure 7:**
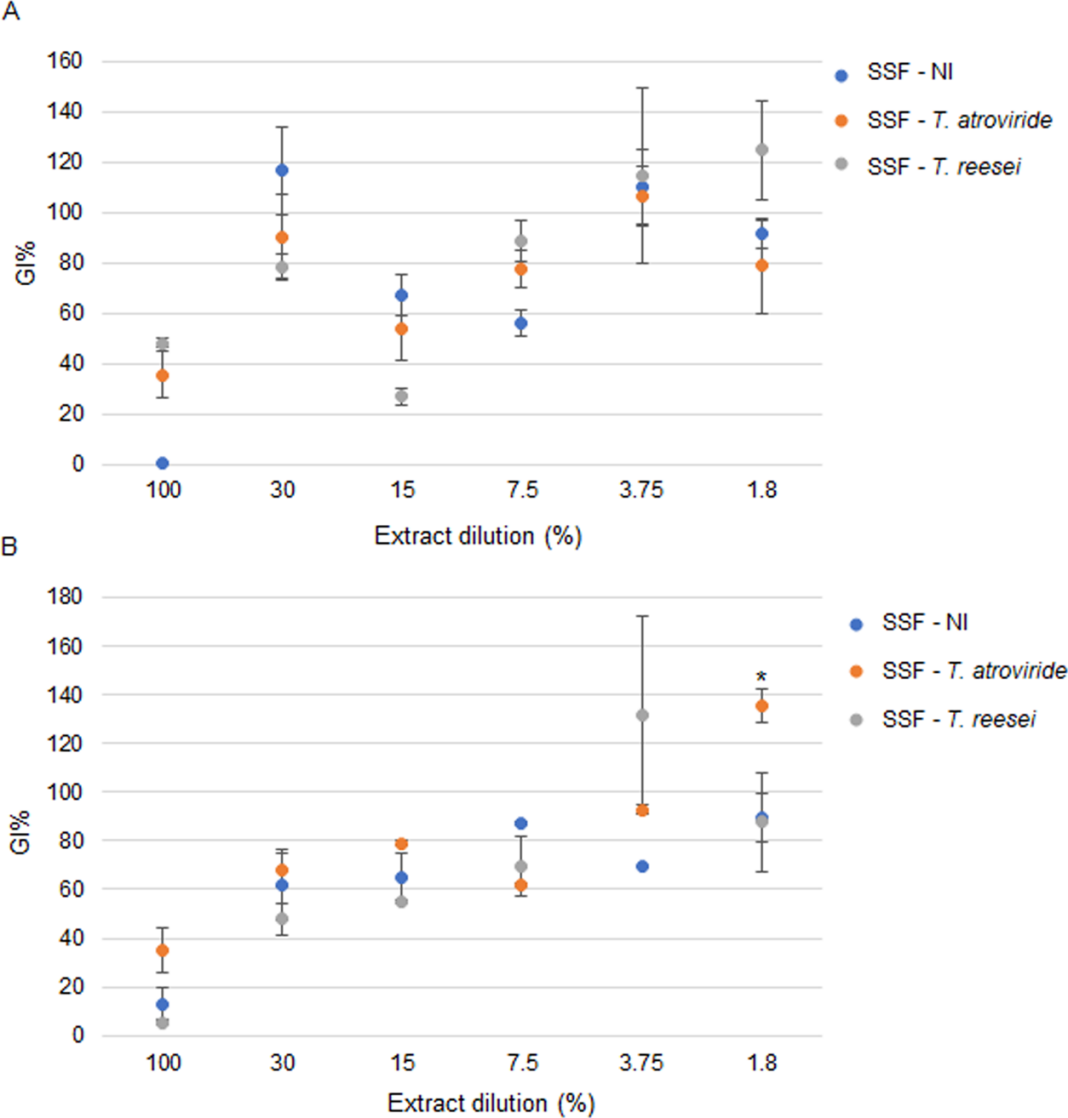
Effects on the germination index (GI) expressed as percentage in cress (*L. sativum*) (A) and in tomato (*S. lycopersicum*) (B) treated with different doses of crude extracts from SSF. Statistically significant vs SSF-NI according to Student’s *t*-test: *p < 0.01.

The germination and the root elongation of *L. sativum* and *S. lycopersicum* were evaluated after seeding on crude extracts of noninoculated (SSF-NI) and *Trichoderma*-fermented substrates at different doses ranging from 100 to 1.8% (Figure 7). The undiluted extracts caused a strong phytotoxicity (germination indexes (GIs) <40%) in both the tested plants. Conversely, the lower dilutions (3.75 and 1.8%) of extracts of the *Trichoderma*-fermented substrates increased the germination of both the plants. In particular, the germination of *S. lycopersicum* was significantly improved by the *T. atroviride*-fermented substrate at 1.8% (GI = 135.5 ± 7.0, *p* < 0.01).

## Discussion

So far, digestate derived from AD for biogas production has been mostly used at farm-scales for improving soils. Commonly, the digestate is separated into solid and liquid phases to better manage the byproduct, to reduce some negative environmental impacts, hence improving the sustainability of the biogas production processes.^32^

As a matter of fact, the liquid fraction of digestate is usually used in agriculture with a high environmental impact. The spray of liquid digestate can result in groundwater contamination,^33^ ammonia volatilization, and nutrient loss in soil, as well as soil contamination with heavy metals and pathogens present in the liquid fraction. Differently, the solid digestate fraction can be easily stored, transported, or converted into biochar, nanocellulose, or used as a biofertilizer. New technologies such as composting and vermicomposting of solid digestate have been applied to limit the environmental impacts and improve a safer application of the digestate in the field.^34^ In addition to these techniques, integrated systems that combine the use of the liquid digestate for microalgae cultivation^35^ and of the solid fraction to yield syngas and pyrochars via thermochemical processes^36^ or by further AD processes to produce biomethane,^35^ bioethanol, and biodiesel have been developed.^37^ In spite of all of these efforts to find new digestate valorizations, very few studies have investigated the possible applications of digestate in fungal biorefineries, and even those are mostly for mushroom cultivation.^38^

The present study focused on the use of the whole digestate (liquid and solid fractions), with a proper addition of other organic wastes, in fungal biorefineries, with the aim to verify this new valorization as a proof of concept. For the first time, it has been demonstrated that the production of various bioproducts—fungal biomass, enzymes, and metabolites of industrial or agricultural interest—is feasible by fungal SSF of the whole digestate.

In the pursuit to avoid the costly and unnecessary separation of solid and liquid phases, sawdust was introduced as a modifier to efficiently manage substrate humidity and porosity, associated with its anoxia. Furthermore, a mixture of organic wastes (fruits) was employed for better fungal growth. An essential parameter for subsequent scale-up of this biotechnological process is the well-characterized profile of the fungal biomass growth throughout the whole life cycle in SSF. To assess the growth kinetics also during the deceleration phase, the period when secondary metabolite production occurs, a prolonged (34 days) time course of fungal growths was conducted. Both species entirely and promptly colonized the substrate, completing their life cycle within 27 days, with a comparable biomass yield albeit produced at slightly different times. *T. reesei* RUT-C30 grew more rapidly than *T. atroviride* Ta13, yet the latter showed a slower growth reduction; both species restarted to grow at the 34th day. A possible explanation of the fungal growth recovery derives from the work of Vrabl^39^ and colleagues demonstrating that with a series of highly standardized bioreactor batch culture experiments, the classical growth curve fails to describe the real growth dynamics of the studied fungi. Accordingly, in this study, during the first growth cycle (27 days), not all of the nutrients might be exhausted and/or the hydrolytic activity of the fungal biomass could have released new nutrients, as supported by the presence of putative polysaccharides identified in the crude extract of *T. reesei* RUT-C30. Thus, the observed last point increase of fungal biomass might be due to the germination of the conidia, produced during the preceding cycle, by virtue of these nutrients. This trend is also supported by the bimodal conidium production previously reported by Daryaei et al.^40^ in *T. atroviride*.

The previous results^16^ about the aptitude of digestate mixed with agro-industrial wastes to serve as a substrate for fungal growth were so confirmed and extended also to the production of enzymes and metabolites.

The genus *Trichoderma* includes a wide number of species in depth characterized for plant growth promotion activity, pathogen biocontrol, and for enzyme production.^41^*Trichoderma* spp. synthesize different multipurpose enzymes such as pectinase, cellulase, chitinase, lipase, protease, amylase, and laccase.^42^ Apart from the well-known chitinase, protease, and microbial cell wall degrading enzymes that improve the biocontrol capabilities (mycoparasitism),^41,43^ also other enzymes could have a role in the biostimulant effects of the *Trichoderma* spp. Prospective impacts of these *Trichoderma* applications include the enhancement of agricultural sustainability, the improvement of agroecosystem equilibrium, and the contribution to green and circular economies. In this frame, the production of enzymes and metabolites in SSF of the whole digestate was addressed. To the best of our knowledge, cellulase and esterase activity is here reported for the first time in SSF of digestate using *Trichoderma* species.

Cellulase is normally produced using different kinds of substrates including lignocellulosic wastes arraying from wheat bran to sewage sludge.^44^ When cellulase is produced by *Trichoderma* in SSF, a wide spectrum of productivity, ranging from 1.5 to 24.9 U/mL, is reported,^43^ but no production was obtained by *T. reesei* SSF of digestate.^45^ Conversely, in this study, a low-cost process, based on SSF of the digestate mixed with agro-food wastes and avoiding biomass pretreatments, supported cellulase production. As expected, *T. reesei* RUT-C30 had the highest cellulase activity at 6 SSF days; in fact, this is a randomly mutagenized strain intensively studied and used for the industrial production of cellulase;^46,47^ still the activity was poor, 0.15 U/mL. As the optimization of SSF as an industrial process for specific enzymatic production was not in the scope of the study, the obtained performances of the process could be improved in the near future.

*T. atroviride* has a moderate cellulase activity, while it is the best esterase producer; this is an interesting enzyme with several industrial applications besides the degradation of natural materials and with a growing demand for. The highest enzyme activities in both *Trichoderma* species were reached at 6 SSF days when the maximum growth was achieved (Figure S5–S6). Esterase and cellulase production in the SSF process seems to be associated with the fungal growth phase, likely due to the metabolism activation of the microorganisms in the presence of a nutrient-rich substrate as previously reported.^48,49^ Although *Trichoderma* spp. have laccase and protease in their enzymatic machinery, the activity of these two enzymes was not observed in the tested conditions.

Other microbial metabolites particularly promising for plant protection against phytopathogens or as biostimulants and growth-promoting agents^50,51^ are the intermediates of the tricarboxylic acid cycle. In this study, organic acids, particularly citric and malic acids, were focused. Citric acid was the most abundant, with the highest concentration detected late in the fermentation process. This could be due to the occurrence of the acidification process of the SSF substrate, which is characterized by alkaline pH values and by a generally high buffer capacity; effectively, the initial substrate pH was 8.5 and decreased to 7.0 after 6 days of fermentation. The other organic acid was malic acid, an intermediate of the citric acid production; it was present only in the early stage of fermentation, and its disappearance during SSF might be connected to the late increase of citric acid.

The two *Trichoderma* spp. strongly differed in their secondary metabolite profile. Interestingly, only *T. atroviride* fermented the digestate-based substrate producing metabolites tentatively identified as gibberellins and oxylipins. These metabolites were identified starting from 6 SSF days, when the analyzed enzyme activities reached their peak; remarkably, the crude extract of *T. atroviride* containing esterase, low amount of cellulase, and two phytohormone-like molecules significantly enhanced the germination and the root elongation compared to the noninoculated substrate. Gibberellins and oxylipins are phytohormones involved in different plant developmental processes and plant defense mechanisms, respectively. Interestingly, these chemical compounds are produced also by fungi to manipulate root development, to colonize, and to induce systemic resistance. Gibberellic acid from fungi has been used in agriculture to enhance plant growth through stem elongation, germination, breaking dormancy, inducing flowering, induction of hydrolytic enzymes, and leaf and fruit senescence in high-value crops. Gibberellins are produced in submerged fermentation by *Gibberella fujikuroi* or *Fusarium verticillioides*, exhibiting low yields with high production costs and broad downstream processing.^9^ The gibberellins present in the crude extract of the SSF substrate fermented by *T. atroviride* were tentatively identified as gibberellin A14 and gibberellin A14 aldehyde. These two molecules are specifically synthetized by fungi,^52^ reported as key intermediates in the production of biological active gibberellins in the fungus *G. fujikuroi.*(53) The poor amount of gibberellins observed in the crude extract before SSF (T0) could be related to fungal biosynthesis during the AD process. Instead, the subsequent gibberellin increase is probably due to *T. atroviride* biosynthesis.

Recently, it was demonstrated that *T. virens* secreted in the maize xylem two oxylipins, which are precursors of jasmonic acid, correlated with induced systemic resistance activation.^31^ To the best of our knowledge, the production of oxylipins by *T. atroviride* in SSF is here reported for the first time. Nevertheless, further studies such integrated NMR and high-resolution MS, are needed for a more accurate identification of these compounds.

Cellulase and citric acid are already produced in larger quantities and in sustainable and effective biotechnological ways; therefore, this SSF process appears more promising for the production of hormone-like molecules. Our findings open the opportunity to produce expensive molecules such as gibberellins and oxylipins through this low-cost SSF process. Oxylipins are molecules already marketed for fruit coloring, for fruit ripening uniformity, and for increasing soluble sugar content; furthermore, it would be interesting to ascertain their activity in the induction of plant systemic resistance against phytopathogens for the potential application in sustainable agriculture. In addition, to avoid expensive purification processes, it could be useful to extend the study of the effects of the crude extract in plant protection as several compounds with this potential activity have been detected.

In summary, the *Trichoderma* spp. SSF of a digestate-based substrate allows the valorization of organic wastes into valuable materials as it provides at the same time (i) a large inoculum of plant growth-promoting fungi, (ii) a mix of bioactive molecules, and (iii) a lower phytotoxicity and enhancement of seed germination.

Our results represent a novel sustainable strategy to produce a potential biostimulant (a cocktail of citric acid, oxylipin, and gibberellin) from the valorization of digestate, in the frame of the EU Bioeconomy Strategy. This biostimulant product would be useful for improving crop growth, resistance to disease, and tolerance to abiotic stresses, paving the way for the new circular application of digestate. Moreover, the *Trichoderma* SSF of digestate mixed with agro-food wastes produces value-added products without generating new wastes. As previously demonstrated,^16^ the digestate-fermented substrate enriched with beneficial fungi is less phytotoxic, and it is able to sustain and enhance seed germination and the root elongation of *L. sativum* and *S. lycopersicum* in a dose- and species-related manner.

## Supporting Information Available

Biomethane plant feeding materials and enzyme activity during fermentation; chromatograms obtained from the UPLC-QTOF analysis of substrates fermented with *Trichoderma* spp.; and overlap of fungal growth curves and enzymes activity in the SSF process (PDF)
