# Supplementary material for: Solid-State Fermentation of Trichoderma spp.: A New Way to Valorize the Agricultural Digestate and Produce Value-Added Bioproducts
Source: J Agric Food Chem. 2023 Feb 3;71(9):3994–4004. doi: 10.1021/acs.jafc.2c07388 (PMC9999421; doi:10.1021/acs.jafc.2c07388)

Support information

Solid-state fermentation of *Trichoderma* spp.: a new way to valorize the agricultural digestate and produce value-added bioproducts

Daniela Bulgari[1 ⁑], Carlotta Alias[1,2 ⁑], Gregorio Peron[3], Giovanni Ribaudo[3], Alessandra Gianoncelli[3], Salvatore Savino[4], Houda Boureghda[5], Zouaoui Bouznad[5], Eugenio Monti[4], Emanuela Gobbi[1]*

[1]Agri-Food and Environmental Microbiology Platform, Department of Molecular and Translational Medicine, University of Brescia, viale Europa, 11, 25123 Brescia, Italy;

[2]B+LabNet-Environmental Sustainability Lab, University of Brescia, Via Branze 45, 25123 Brescia, Italy;

[3]Proteomics Platform, AgroFood Lab, Department of Molecular and Translational Medicine, University of Brescia, viale Europa, 11, 25123, Brescia, Italy

[4]Unit of Biotechnology, Department of Molecular and Translational Medicine, University of Brescia, viale Europa 11, Brescia 25123, Italy

[5]Department of Botany, Laboratory of Phytopathology and Molecular Biology, Ecole Nationale Supérieure Agronomique (ENSA), El Harrach, 16200, Algeria

⁑ Co-first author

Corresponding author

Emanuela Gobbi

Address: viale Europa, 11, 25123, Brescia, Italy

E-mail: emanuela.gobbi@unibs.it

Table S1. Biomethane plant AD feedstock materials

Constituents	% (w/w)
Dairy cattle slurry	38.1
Triticale	24.5
Silage corn stalks	13.9
Silage corn (1st harvest)	13.7
Silage corn (2nd harvest)	5.5
Fresh cattle manure	4.3

Table S2. Cellulase and esterase activity in *Trichoderma reesei* and *Trichoderma atroviride* during solid state fermentation

Fermentation time		Cellulase (mU/g)		Esterase (mU/g)	
**Sampling points**	**Days**	***T. reesei***	***T. atroviride***	***T. reesei***	***T. atroviride***
T0	0	0	0	5.6 ± 1.3	3.5 ± 0.4
T1	3	91.3 ± 4.9	83.3 ± 1.6	11.7 ± 2.6	73.3 ± 0.6
T2	6	219.1 ± 3.3	49.5 ± 4.6	113.9± 11.9	163.1 ± 7.3
T3	13	140.2 ± 31.3	33.86 ± 2.3	25.8 ± 20.9	123.8 ± 5.6
T4	20	116.7 ± 8.1	36.8 ± 4.3	14.6 ± 0.6	81.3 ± 5.3
T5	27	142.3 ± 8.9	33.4 ± 3.1	53.5 ± 4.0	77.7 ± 1.0
T6	34	58.9 ± 3.4	54.7 ± 2.1	4.4 ± 1.3	57.7 ± 2.6

Figure S1. Chromatograms obtained from the UPLC-QTOF analysis of crude extract of *T. reesei* RUT-C30 solid state fermentation.

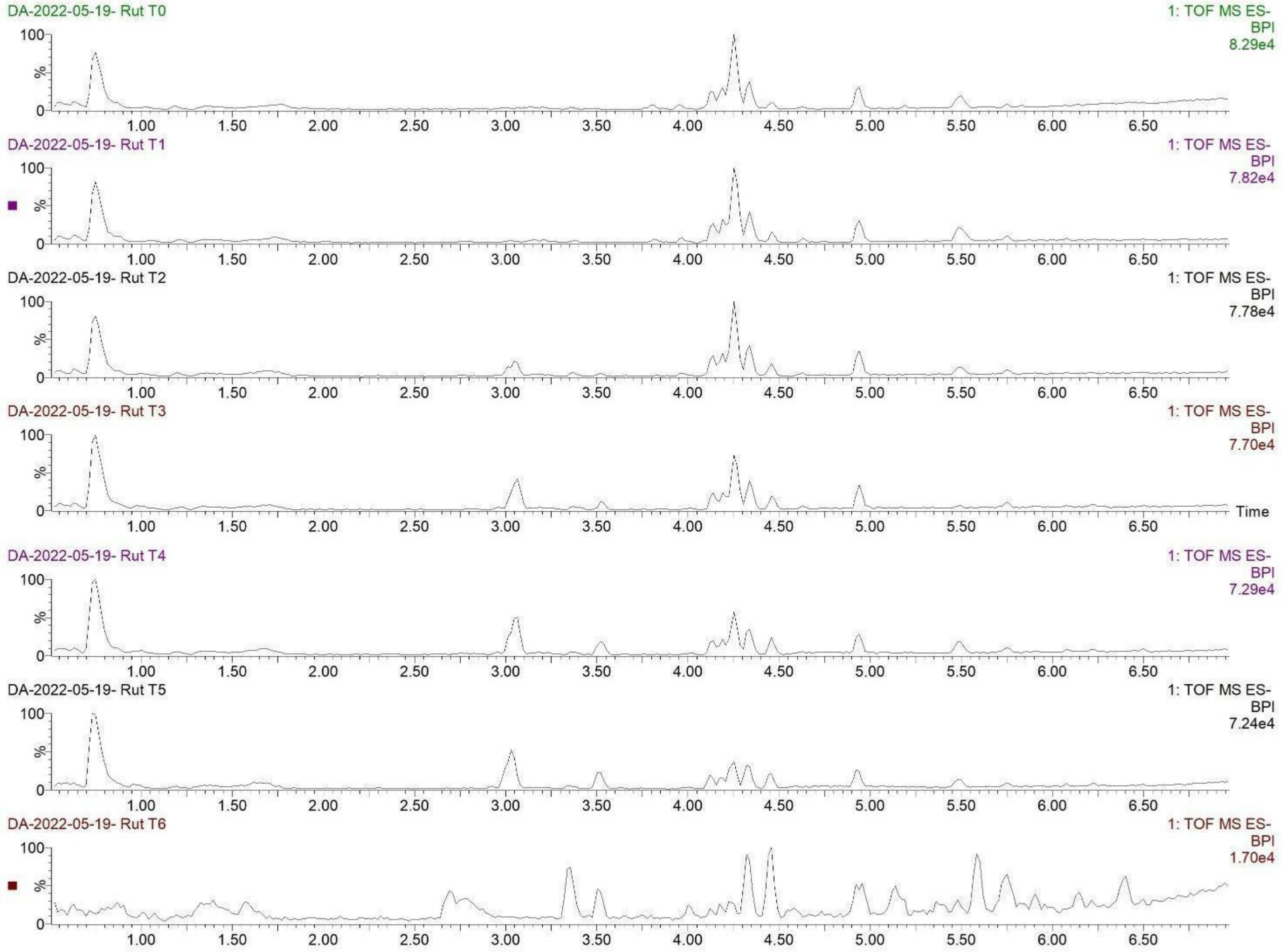

Figure S2. Chromatograms obtained from the UPLC-QTOF analysis of crude extract of *T. atroviride* Ta13 solid state fermentation.

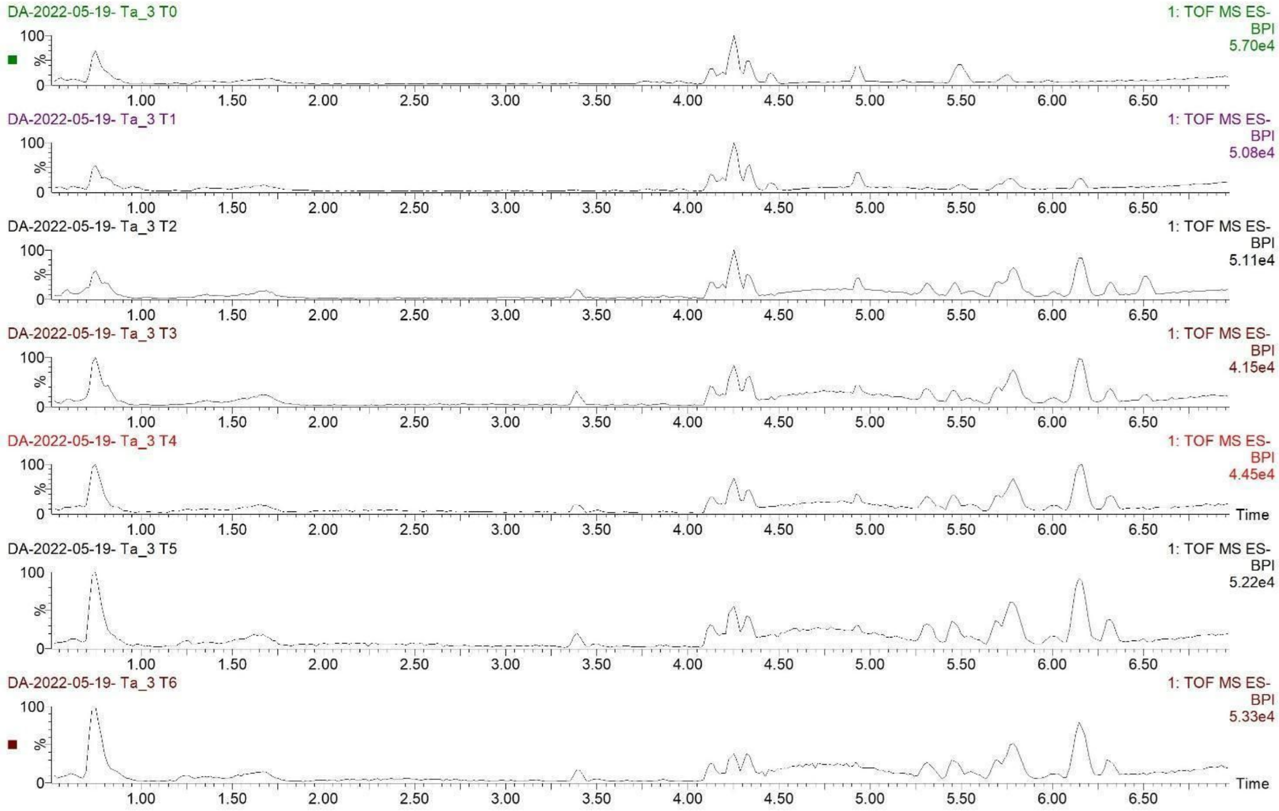

Figure S3. Chromatograms obtained from the UPLC-QTOF analysis of crude extract from non-inoculated samples at T6 of solid state fermentation.

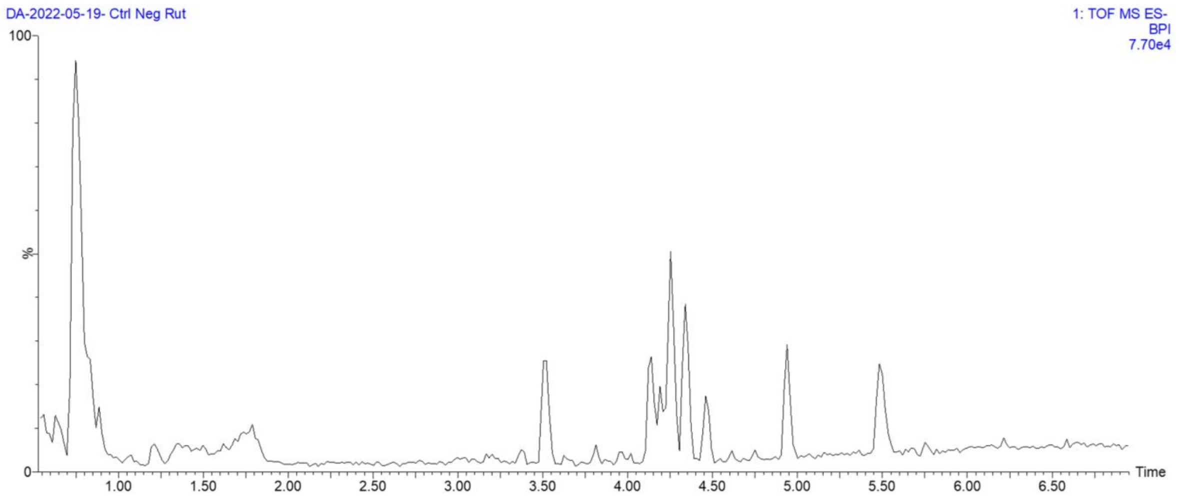

Figure S4. MS spectra of the molecular species eluted at 6.15 min in the UPLC-QTOF analysis of the substrate fermented with *T. atroviride*. The metabolite was tentatively identified as oxylipin (polyhydroxylated fatty acid). Panel A shows the full-scan spectrum of the molecular ion, m/z 349.2025 ($C_{20}H_{30}O_5$) and Panel B shows the spectrum of the fragments at m/z 331.1907 and at m/z 331.1974.

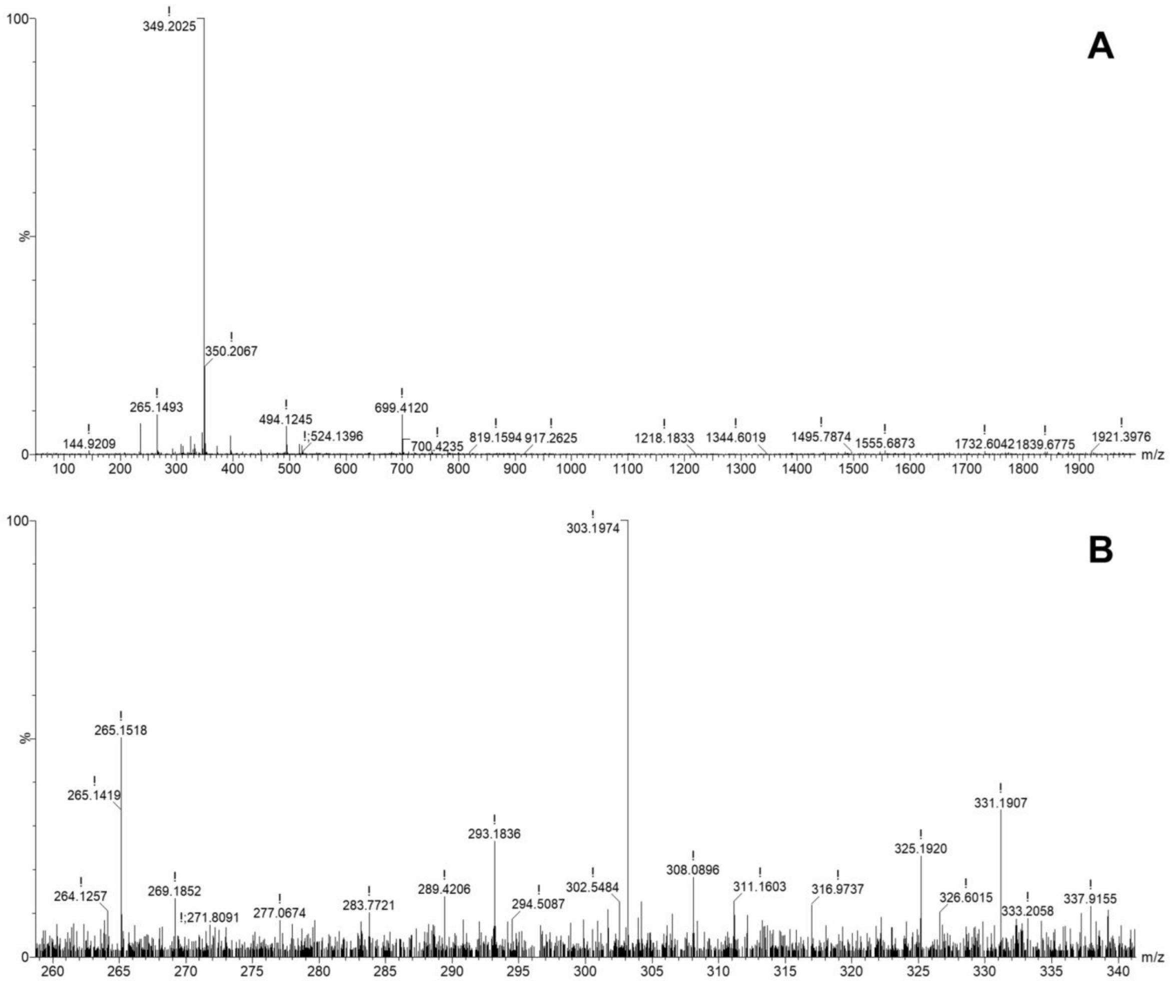

Figure S5. Combined cellulase activity and fungal growth, measured for the entire 70g substrate present in each box, for (a) *Trichoderma reesei* RUT-C30 and (b) *Trichoderma atroviride* Ta13

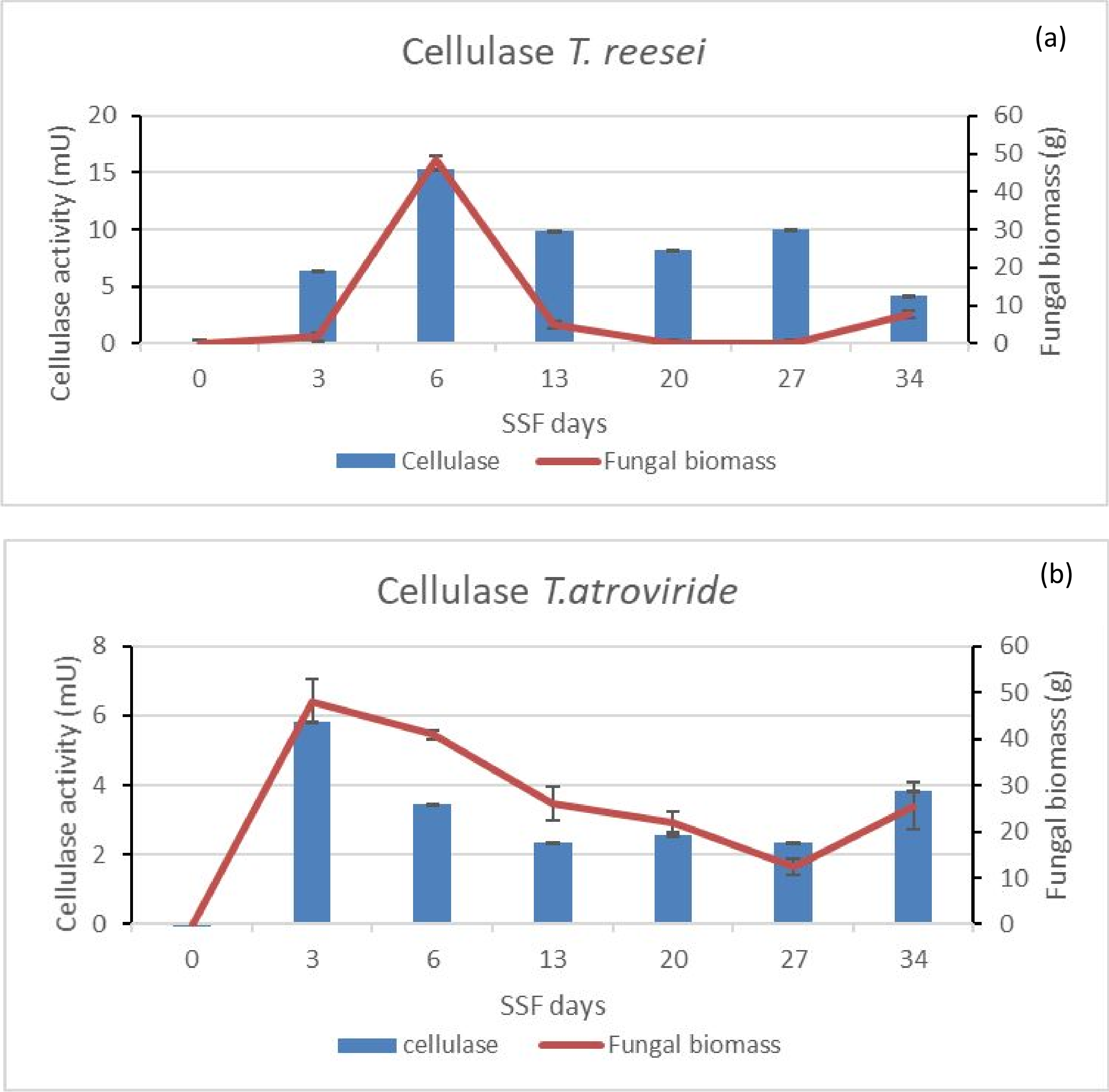

Figure S6. Combined esterase activity and fungal growth, measured for the entire 70g substrate present in each box, for (a) *Trichoderma reesei* RUT-C30 and (b) *Trichoderma atroviride* Ta13.

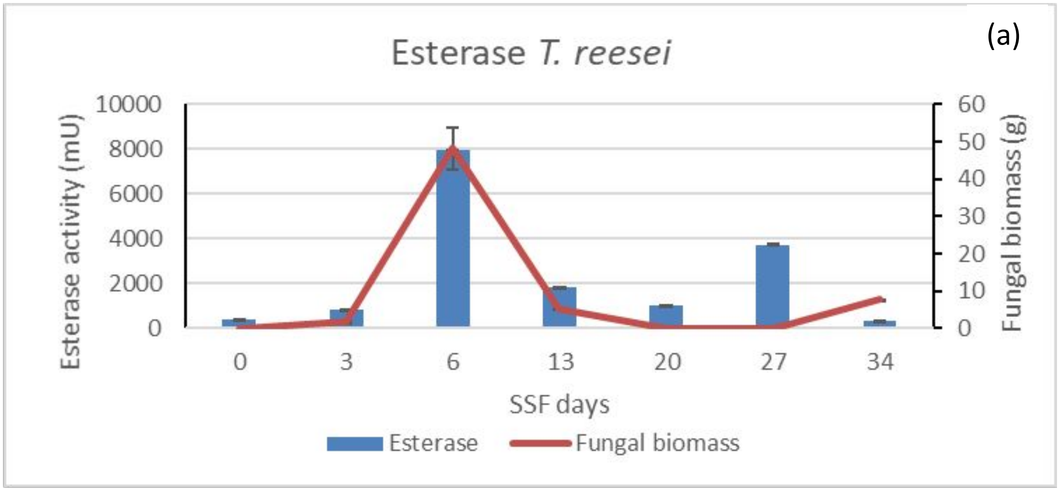

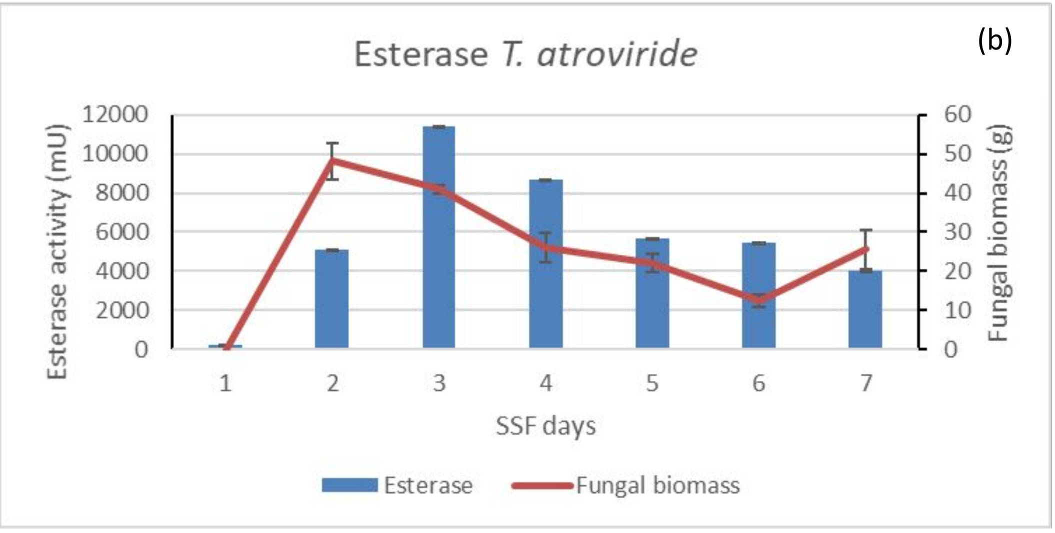

Supplement: Supplementary file 1 — jf2c07388_si_001.pdf [file jf2c07388_si_001.pdf]
